# Diagnosing Preclinical Cardiac Dysfunction in Swiss Childhood Cancer Survivors: Protocol for a Single-Center Cohort Study

**DOI:** 10.2196/17724

**Published:** 2020-06-10

**Authors:** Christina Schindera, Claudia Elisabeth Kuehni, Mladen Pavlovic, Eva Simona Haegler-Laube, Daniel Rhyner, Nicolas Waespe, Jochen Roessler, Thomas Suter, Nicolas Xavier von der Weid

**Affiliations:** 1 Childhood Cancer Registry Institute of Social and Preventive Medicine University of Bern Bern Switzerland; 2 Pediatric Oncology/Hematology University Children's Hospital Basel University of Basel Basel Switzerland; 3 Pediatric Hematology and Oncology University Children’s Hospital Bern University of Bern Bern Switzerland; 4 Department of Cardiology Inselspital, Bern University Hospital University of Bern Bern Switzerland; 5 Platform of Pediatric Onco-Hematology research, CANSEARCH Laboratory Department of Pediatrics, Gynecology, and Obstetrics University of Geneva Geneva Switzerland

**Keywords:** cardiotoxicity, Switzerland, echocardiography, speckle tracking, strain, anthracyclines, alkylating agents, steroids, cardiac radiation

## Abstract

**Background:**

Cardiovascular disease is the leading nonmalignant cause of late deaths in childhood cancer survivors. Cardiovascular disease and cardiac dysfunction can remain asymptomatic for many years, but eventually lead to progressive disease with high morbidity and mortality. Early detection and intervention are therefore crucial to improve outcomes.

**Objective:**

In our study, we aim to assess the prevalence of preclinical cardiac dysfunction in adult childhood cancer survivors using conventional and speckle tracking echocardiography; determine the association between cardiac dysfunction and treatment-related risk factors (anthracyclines, alkylating agents, steroids, cardiac radiation) and modifiable cardiovascular risk factors (abdominal obesity, hypertension); investigate the development of cardiac dysfunction longitudinally in a defined cohort; study the association between cardiac dysfunction and other health outcomes like pulmonary disease, endocrine disease, renal disease, quality of life, fatigue, strength and endurance, and physical activity; and gain experience conducting a clinical study of childhood cancer survivors that will be extended to a national, multicenter study of cardiac complications.

**Methods:**

For this retrospective cohort study, we will invite ≥5-year childhood cancer survivors who were treated at the University Children's Hospital Bern, Switzerland with any chemotherapy or cardiac radiation since 1976 and who are ≥18 years of age at the time of the study for a cardiac assessment at the University Hospital Bern. This includes 544 childhood cancer survivors, of whom about half were treated with anthracyclines and/or cardiac radiation and half with any other chemotherapy. The standardized cardiac assessment includes a medical history focusing on signs of cardiovascular disease and its risk factors, a physical examination, anthropometry, vital parameters, the 1-minute sit-to-stand test, and echocardiography including 2-dimensional speckle tracking.

**Results:**

We will invite 544 eligible childhood cancer survivors (median age at the time of the study, 32.5 years; median length of time since diagnosis, 25.0 years) for a cardiac assessment. Of these survivors, 300 (55%) are at high risk, and 244 (45%) are at standard risk of cardiac dysfunction.

**Conclusions:**

This study will determine the prevalence of preclinical cardiac dysfunction in Swiss childhood cancer survivors, inform whether speckle tracking echocardiography is more sensitive to cardiac dysfunction than conventional echocardiography, and give a detailed picture of risk factors for cardiac dysfunction. The results will help improve primary treatment and follow-up care of children with cancer.

**Trial Registration:**

ClinicalTrials.gov NCT03790943; https://clinicaltrials.gov/ct2/show/NCT03790943

**International Registered Report Identifier (IRRID):**

DERR1-10.2196/17724

## Introduction

Survival of childhood cancer has improved, and the number of childhood cancer survivors (CCS) has greatly increased during recent decades [1,2]. Consequently, more survivors face increased long-term morbidity and mortality due to chronic health conditions such as cardiovascular disease, pulmonary disease, and secondary neoplasms [3-5]. Among these, cardiovascular disease is the leading nonmalignant cause of death among CCS [3] with a cumulative incidence that increases up to 30 years after cancer diagnosis [6]. Heart failure, myocardial infarction, pericardial and valvular disease, and arrhythmias are all associated with treatments used in childhood cancer patients.

Studies from North America, Germany, and The Netherlands have assessed survivors exposed to cardiotoxic cancer therapy, in whom a prevalence of subclinical cardiac dysfunction ranging from 6% to 27% was identified via conventional echocardiography [7-10]. This suggests that many CCS have impaired cardiac function that might progress to clinical heart failure later in life. The North American study also found that a further 32% of survivors with otherwise normal conventional echocardiography showed evidence of cardiac dysfunction with abnormal strain measurements by speckle tracking echocardiography, a novel echocardiographic technique [7]. Additional studies have also suggested that speckle tracking echocardiography might be more sensitive to preclinical cardiac dysfunction than conventional echocardiography in CCS [11,12].

Most studies have assessed survivors exposed to anthracyclines and cardiac radiation, which are the most important treatment-related risk factors [13]. Yet, other treatments may also increase the risk of cardiac dysfunction in CCS. Survivors who were not exposed to anthracyclines or cardiac radiation have demonstrated decreased left ventricular (LV) mass and increased cardiac biomarkers compared to siblings [14].

The North American Childhood Cancer Survivor Study analyzed self-reported data on cardiovascular risk factors in more than 10,000 adult CCS and showed that hypertension alone and in combination with other modifiable cardiovascular risk factors significantly increased the risk for heart failure, coronary artery disease, valvular disease, and arrhythmia in adult CCS [15]. The likelihood that modifiable cardiovascular risk factors might potentiate the increased risk of treatment-related cardiovascular disease in CCS thus motivates this study of cardiac dysfunction in adult CCS.

## Methods

### Study Objectives

The first and primary objective of this study is to assess the prevalence of preclinical cardiac dysfunction in adult CCS using 2-dimensional (2D) and 3-dimensional (3D) conventional and 2D speckle tracking echocardiography. Second, we will determine the association between cardiac dysfunction and the risk related with treatment (anthracyclines, alkylating agents, steroids, and cardiac radiation) as well as the modifiable cardiovascular risk factors abdominal obesity and hypertension. Our third objective is to investigate the development of cardiac dysfunction longitudinally in a defined cohort. Fourth, we will study the association between cardiac dysfunction and other health outcomes like pulmonary, endocrine, and renal diseases; quality of life; fatigue; strength and endurance; and physical activity. Finally, pursuing these objectives will provide experience conducting a clinical study of CCS that could be used for a national, multicenter study of cardiac complications.

### Primary Outcome

The primary outcomes of this study are abnormal 2D and 3D LV ejection fraction (LVEF) measured using conventional echocardiography and abnormal global longitudinal strain (GLS) measured using 2D speckle tracking echocardiography (Textbox 1).

**Components of cardiac assessment collected for childhood cancer survivors.**

**Conventional echocardiography**

- Left ventricular (LV) systolic function
  2-dimensional (2D) and 3-dimensional (3D) LV ejection fraction (LVEF)
- LV diastolic function
  Early diastolic LV filling velocity (E)
  Late diastolic LV filling velocity (A)
  Early to late LV filling velocity (E/A ratio)
  Mitral annular early diastolic velocity (e’) (septal and lateral)
  Peak mitral flow velocity (E/e’ ratio)
  Peak tricuspid regurgitation (TR) velocity
  Left atrial (LA) maximum volume index
- Right atrium (RA), right ventricle (RV), RV/RA ratio
- Valvular dysfunction, respiratory variation, size of the vena cava

**2D speckle tracking echocardiography**

- LV systolic function
  Global longitudinal strain (GLS)
  Global circumferential strain (GCS)
  Global radial strain (GRS)

**Personal history**

- Demographic and socioeconomic characteristics
- Clinical characteristics
  Cardiac symptoms
  History of cardiovascular disease
  Modifiable cardiovascular risk factors
  Chronic conditions
  Sleeping habits
  Medications
  Thoracic surgeries
  Family history of cardiovascular disease and risk factors
- Pictorial images for perception of weight status
- Qualitative questions

**Anthropometry and blood pressure**

- Weight and height
- Waist and hip circumference
- Blood pressure

**Physical examination**

- Auscultation of the heart and lungs
- Palpation of pulses
  Carotid
  Radial
  Tibial
  Dorsal feet
- Signs of heart failure
  Jugular vein pressure
  Hepato-jugular reflux
  Edema of the lower extremities
  Size of the liver and spleen
  Documentation of thoracic scars

**1-minute sit-to-stand test**

**Counselling of survivors and medical letter**

**Online questionnaires**

### Secondary Outcomes

Secondary outcomes are other conventional echocardiographic parameters of abnormal LV diastolic function (early diastolic LV filling velocity [E], late diastolic LV filling velocity [A], early to late LV filling velocity [E/A ratio], mitral annular early diastolic velocity [e’], peak mitral flow velocity [E/e’ ratio], peak tricuspid regurgitation [TR] velocity, left atrial [LA] maximum volume index), right atrium [RA], right ventricle [RV], RV/RA ratio, valvular dysfunction, respiratory variation, and size of the vena cava, and speckle tracking echocardiography–derived parameters of abnormal LV systolic function (global circumferential strain [GCS], global radial strain [GRS]). Other secondary outcomes include impaired quality of life and fatigue.

### Intermediate Outcomes and Exposures

We also collect information about treatment with anthracyclines, alkylating agents, steroids, and cardiac radiation; modifiable cardiovascular risk factors (abdominal obesity and hypertension); and other health outcomes including pulmonary, endocrine, and renal diseases; strength and endurance; and physical activity.

### Study Design, Study Population, and Inclusion Criteria

**Figure 1:**
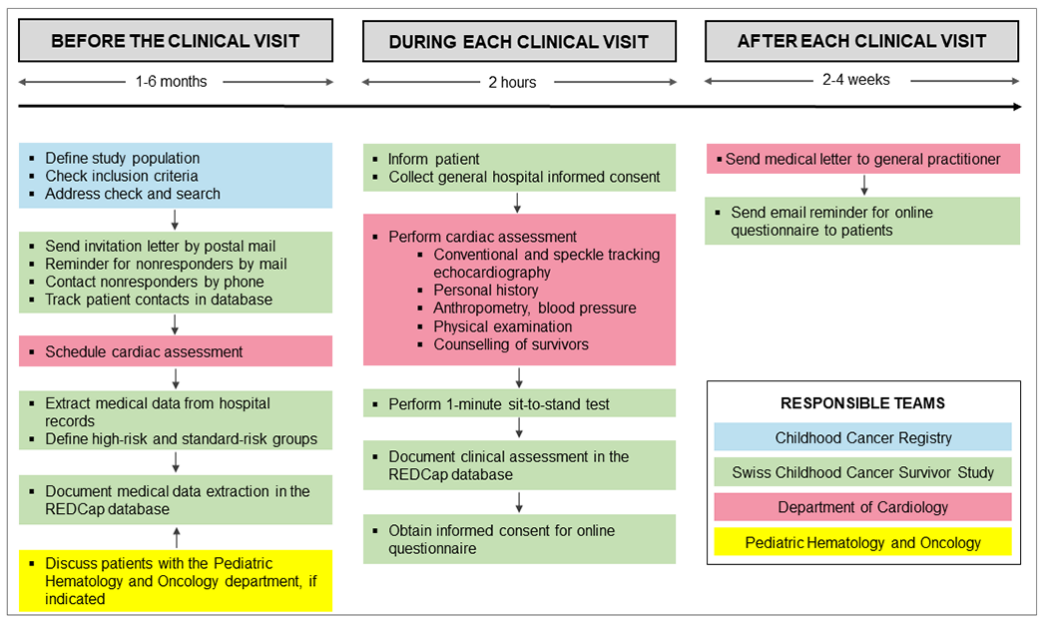
Responsible teams in the study of preclinical diagnosis of cardiac dysfunction in childhood cancer survivors.

This retrospective cohort study is part of routine clinical follow-up care and a collaborative and interdisciplinary effort of the Childhood Cancer Registry, Swiss Childhood Cancer Survivor Study, and departments of Pediatric Hematology and Oncology and Pediatric and Adult Cardiology at the University Hospital Bern, Switzerland (Textbox 2, Figure 1). The study includes all ≥5 year CCS diagnosed with childhood cancer starting in 1976, treated at the University Children’s Hospital Bern in Switzerland with any chemotherapy and/or cardiac radiation, aged ≥18 years at the time of the study, and registered in the Childhood Cancer Registry. The registry includes all patients in Switzerland diagnosed at age 0-20 years with leukemia, lymphoma, central nervous system tumors, malignant solid tumors, or Langerhans cell histiocytosis [16]. We classify cancer diagnoses according to the International Classification of Childhood Cancer, third edition into 12 main groups [17] and Langerhans cell histiocytosis. Recent estimates indicate that the registry includes >95% of children diagnosed at <16 years since 1995 in Switzerland [18]. We exclude survivors who were treated with surgery only and/or radiation other than cardiac radiation because these survivors have a low risk of developing cardiac dysfunction. Ethics approval of this study was granted by the Ethics Committee of the Canton of Bern, Switzerland (KEK-BE: 2017-01612), and the study is registered at ClinicalTrials.gov (identifier: NCT03790943). Informed consent, as documented with a signature, is obtained from each survivor prior to participation in the study.

**Teams and staff members involved in the workflow of the study of preclinical diagnosis of cardiac dysfunction.**

**Childhood Cancer Registry**

- Administrative staff

**Swiss Childhood Cancer Survivor Study**

- PhD student
- Study nurse
- Master students

**Pediatric Hematology and Oncology**

- Head of pediatric hematology and oncology

**Department of Cardiology**

- Cardiologists specialized in echocardiography
- Cardiologist specialized in cardio-oncology
- Nurse practitioner specialized in cardio-oncology
- Administrative staff

### Study Logistics

Current addresses of eligible survivors are obtained from the Childhood Cancer Registry and updated via the Swiss postal service where necessary (Figure 1). We send an invitation letter to survivors explaining why a cardiac assessment is useful, and it also describes the planned examinations and visit location in the Department of Cardiology at the University Hospital Bern. Survivors are asked to return a response form indicating their interest in participation in the study and the date and place of previous cardiac assessment(s). Nonresponders receive up to two reminders by mail before we try to contact them by phone. The administrative personnel of the Department of Cardiology schedules an appointment for a cardiac assessment via mail with survivors who agree to participate. The study is part of the routine follow-up care offered to CCS and is paid for by health insurance. All patient contacts are documented in a patient-tracking database.

### Medical Data Extraction

We extract the following data on each survivor from the cancer registry: cancer diagnose(s), relapse(s), age at cancer diagnosis, year of cancer diagnosis, and whether the person had chemotherapy, radiation (if so, the location of radiation), surgery, or hematopoietic stem cell transplantation (Figure 1).

We collect cumulative doses of anthracyclines, steroids, and alkylating agents from medical records (Textbox 3). We record patient weight, patient height, and the doses in each chemotherapy cycle. We calculate the cumulative doses per unit body surface area, expressed in milligrams or grams per square meter (Textbox 3).

Cardiac radiation includes different radiation fields [19] and is collected from medical records (Textbox 3). We use the maximum documented dose of the field involving the heart and add the dose of total body irradiation.

**Cumulative doses of chemotherapy and cardiac radiation extracted from medical records.**

**Cumulative doses of chemotherapy**

- Anthracyclines with doxorubicin-equivalent doses (mg/m^2^) [19]
  Doxorubicin x 1.0
  Daunorubicin x 0.5
  Epirubicin x 0.67
  Idarubicin x 5.0
  Mitoxantrone x 4.0
- Alkylating agents with cyclophosphamide-equivalent doses (mg/m^2^) [20]
  Cyclophosphamide x 1.0
  Ifosfamide x 4.09
- Steroids with prednisone-equivalent doses (g/m^2^) [21]
  Prednisone x 1.0
  Dexamethasone x 6.67

**Cumulative doses of cardiac radiation** [19]

- Cardiac radiation (gray)
  Chest
  Abdomen
  Whole or thoracic spine
  Total body irradiation

### Definition of High-Risk and Standard-Risk Groups

**Figure 2:**
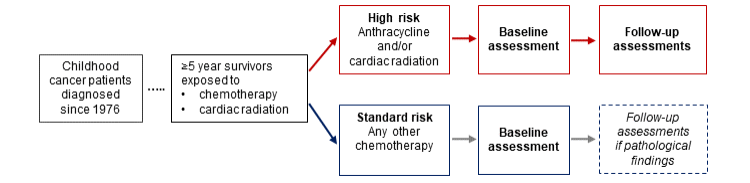
Study design and risk group stratification of childhood cancer survivors.

Patients with exposure to any cumulative dose of anthracyclines and/or cardiac radiation (chest, abdomen, whole or thoracic spine, total body irradiation) are placed in the high-risk group (Figure 2). High-risk patients are assessed longitudinally with baseline and follow-up cardiac assessments according to the Children’s Oncology Group Long-Term Follow-Up Guidelines, Version 5.0, October 2018 [19]. Survivors with exposure to any chemotherapy other than anthracyclines are assigned to a standard-risk group and evaluated cross-sectionally unless a cardiac follow-up assessment is clinically indicated. All survivors with surgery only or radiation other than cardiac are excluded from this study and are seen only within the routine follow-up care without echocardiography.

### Patient Information and Informed Consent

At the cardiac assessment, we give survivors oral and written information about the clinical study (Figure 1). The general hospital informed consent form that participants sign is a standard consent form widely used in Swiss inpatient and outpatient settings to enable research with clinical data.

### Echocardiography and Definition of Cardiac Dysfunction

Echocardiography is performed by experienced cardiologists from the Department of Cardiology who are blinded with respect to the patient’s cancer treatment and risk group (Textbox 2). Conventional echocardiography includes assessment of LV systolic function (2D and 3D LVEF), LV diastolic function (E, A, E/A ratio, septal and lateral e’, E/e’ ratio, peak TR velocity, LA maximum volume index), RA, RV, RV/RA ratio, valvular dysfunction, respiratory variation, and size of the vena cava (Textbox 1, Figure 1) using a GE Vivid E9 or E95 (GE Vingmed, Horten, Norway). 2D speckle tracking echocardiography includes GLS, GCS, and GRS and is performed using vendor-independent software (Tomtec Imaging Systems, Unterschleissheim, Germany).

We define cardiac dysfunction according to the American Society of Echocardiography and European Association of Cardiovascular Imaging recommendations [22,23].

LV systolic dysfunction is defined as 2D/3D LVEF <52% for men and <54% for women [22].

The LV diastolic dysfunction definition depends on whether a patient has normal or impaired LVEF [23]. In patients with normal LVEF, four parameters and cutoff levels are used: (1) E/e´ ratio >14, (2) septal e´ velocity <7 cm/s or lateral e´ velocity <10 cm/s, (3) TR velocity >2.8 m/s, and (4) LA volume index >34 mL/m². Diastolic function is defined as abnormal if more than half of available parameters meet the cutoff levels, as normal if more than half of available parameters do not meet cutoff levels, and as inconclusive if half of the parameters do not meet the cutoff levels. In patients with impaired LVEF, the E/A ratio is used for stratification into three grades of diastolic dysfunction. An E/A ratio ≤0.8 and a peak mitral flow velocity E ≤50 cm/sec are defined as grade I diastolic dysfunction. An E/A ratio ≥2.0 is defined as grade III diastolic dysfunction. If the E/A ratio is ≤0.8 and peak mitral flow velocity E is >50 cm/sec, or E/A >0.8 but <2, three additional parameters and cutoff values are used: (1) peak TR velocity >2.8 m/sec, (2) E/e´ ratio >14, and (3) LA maximum volume index >34 mL/m². Grade II diastolic dysfunction is present if more than half of parameters meet the cutoff values, grade I diastolic dysfunction is present if only one available parameter meets the cutoff levels, and the study is inconclusive if only one parameter is available or in case of 50% discordance [23].

Abnormal strain (GLS, GCS, GRS) is defined as >2 SD below the mean using sex-specific, age-specific, vendor-specific, and software-specific strain values [24].

### Personal History

We take a comprehensive personal history of survivors' demographic and socioeconomic characteristics: current occupation, employment status, work hours per week, marital status, offspring, and housing situation (Textbox 1, Figure 1). We also obtain clinical characteristics including cardiac symptoms (New York Heart Association class I-IV), history of cardiovascular disease, modifiable cardiovascular risk factors (hypertension, diabetes, dyslipidemia, smoking, physical inactivity, drug consumption), chronic conditions (pulmonary, endocrine, and renal diseases), sleeping habits, medications, thoracic surgeries, and family history of cardiovascular diseases and risk factors. To determine perception of weight status, we use pictorial images of women and men similar to Harris et al [25] and ask the patient to indicate the picture that best matches the weight status of his or her parents, siblings, and the patient’s own weight. We also ask patients: “Was it a big effort for you to come to the hospital for today’s appointment, does your history of childhood cancer play a role in your daily life, and are you afraid that your treatment for cancer during childhood caused any medical problems in adulthood?” We also ask patients if they have any questions, requests, or wishes to direct to us.

### Anthropometry and Blood Pressure

Weight and standing height are measured using standard procedures, while the patient is barefoot and in light clothes (Textbox 1, Figure 1). Weight is determined to the nearest 0.1 kg and height to the nearest 0.5 cm. BMI is expressed as kg/m^2^ [26]. Waist and hip circumferences are measured using a measuring tape to the nearest 0.1 cm. Waist circumference is measured at the midpoint between the lower margin of the lowest rib and the top of the iliac crest, and hip circumference is measured at the widest circumference over the buttocks [26]. The waist-hip ratio is calculated as waist circumference divided by hip circumference. Blood pressure is measured comfortably in a quiet environment with three measurements repeated in a sitting position, and the average of the last two readings is recorded. Additional measurements are taken if the first two readings of systolic or diastolic blood pressure differ by >10 mm Hg [27].

### Physical Examination

We perform a thorough physical examination with special emphasis on signs of cardiovascular disease (Textbox 1, Figure 1). This includes auscultation of the heart and lungs; palpation of the carotid, radial, tibial, and dorsal foot artery pulses; and examination of the jugular vein pressure, hepato-jugular reflux, edema of the lower extremities, size of the liver and spleen, and documentation of thoracic scars.

### 1-Minute Sit-to-Stand Test

We have the patient perform the 1-minute sit-to-stand test (STS), which captures the number of times a person can stand up and sit down on a regular chair in 1 minute (Textbox 1, Figure 1) [28]. The STS is an estimate of lower body muscular strength and endurance. We compare our population with population-based, age-adjusted, and sex-adjusted Swiss reference values [28].

### Counselling of Survivors and the Medical Letter

At the end of the cardiac assessment, a cardiologist specialized in cardio-oncology and who did not perform the echocardiography explains the results of the echocardiography to survivors and counsels them on their cardiac function and the presence (or absence) of modifiable cardiovascular risk factors (Textbox 1, Figure 1). Recommendations on follow-up assessments are based on the Children’s Oncology Group Long-Term Follow-Up Guidelines, Version 5.0, October 2018 [19]. A medical letter summing up the results of the cardiac assessment is sent to the survivor’s general practitioner.

### Online Questionnaire

We ask survivors to complete 4 questionnaires after returning home. The Short Form 36 Health Survey assesses health-related quality of life [29] and has been used before in CCS [30]. The Seven-Day Physical Activity Recall questionnaire measures moderate physical activity, vigorous physical activity, and sleep during the last 7 days [31]. Fatigue is assessed using the Checklist Individual Strength questionnaire, a validated 20-item questionnaire that identifies different aspects of fatigue within the previous 2 weeks [32]. Diet and alcohol consumption are obtained using questions from the Swiss Childhood Cancer Survivor Study questionnaire [33]. We ask survivors to provide separate informed consent for the online questionnaires. Survivors not completing the online questionnaires within 2 weeks after the clinical visit are reminded by email.

### Documentation

All parts of the cardiac assessment are directly entered into a dedicated REDCap (version 8.5.19, Vanderbilt University, Nashville, TN) database to minimize risk of disclosure. Within the database, each survivor has a unique ID. No personal information can be obtained with this number. Data containing survivors’ unique IDs are stored on encrypted devices or secured servers at the University of Bern.

### Cardiac Assessment, Phase 1

**Figure 3:**
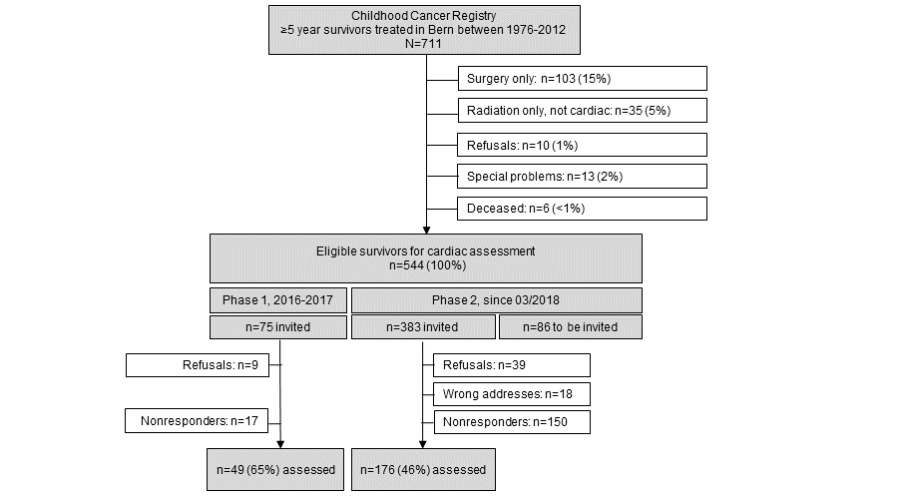
Recruitment of childhood cancer survivors eligible for the study, current as of October 1, 2019.

In 2016-2017, we started using standardized echocardiography for cardiac assessment, as already described (Figure 3). At that time, personal history, blood pressure, physical examination, and the STS were not yet assessed in a standardized way. The personal history and online questionnaires were retrospectively completed by phone interviews between May 2017 and June 2017.

### Cardiac Assessment, Phase 2

Since March 2018, we have been collecting all data in a standardized way. Data collection is performed by the study team from the Childhood Cancer Registry, Swiss Childhood Cancer Survivor Study, Department of Pediatric Hematology and Oncology at the University Hospital Bern, and Department of Cardiology at the University Hospital Bern (Textbox 2, Figure 3).

### Statistical Analyses and Power Calculations

We will compare characteristics of responders and nonresponders using chi-square tests and perform univariable and multivariable logistic regression analyses to investigate the association between cardiotoxic treatment exposures (anthracyclines, alkylating agents, steroids, cardiac radiation) and modifiable cardiovascular risk factors (abdominal obesity, hypertension) and cardiac dysfunction adjusting for sex, age at study, and follow-up time.

From our eligible study population of 544 CSS and based on an expected response rate of 65%, we estimate that approximately 354 survivors will attend the cardiac assessment (Figure 3). Among these participants, about 55% (195/354) are at high risk, and 45% (159/354) are at standard risk for cardiac dysfunction. These numbers will provide a power of 80% and α of .05 to detect a significant difference in cardiac dysfunction in high-risk survivors, assuming 6% cardiac dysfunction for LVEF and 32% for GLS [7]. In standard-risk survivors, the prevalences of abnormal LVEF and GLS will be lower; therefore, the sample size might be borderline sufficient. Considering that we plan to extend this single-center cohort study to a multicenter study, numbers will substantially increase.

We will use STATA software (Version 15.1, Stata Corporation, Austin, TX) for statistical analyses.

## Results

On January 1, 2018, the Childhood Cancer Registry included 711 survivors aged ≥18 years who had been diagnosed and treated at the University Children’s Hospital Bern since 1976 and had survived ≥5 years (Figure 3). Among those, 103 were excluded because of surgery as the only treatment, 35 survivors because of radiation only other than cardiac, and 29 survivors because they did not want to be contacted, had specific problems (eg, did not want to be invited for clinical studies because of emotional stress), or had died. Among the remaining survivors, 544 met the inclusion criteria for an invitation to a clinical visit (Table 1, Figure 3). This number includes 300 survivors (300/544, 55%) at high risk for cardiac dysfunction and 244 survivors (244/544, 45%) at standard risk for cardiac dysfunction, with a median age at the time of the study of 32.5 years and a median time since diagnosis of 25.0 years (Table 1). In Phase 1 (2016-2017), 75 survivors were invited, and 49 survivors attended the cardiac assessment, for a response rate of 65%; phase 2 is ongoing (Figure 3). We plan to recruit new 5-year survivors continuously into the study, so the size of the cohort will increase and numbers will change.

**Table 1:** Demographic and clinical characteristics of survivors eligible for participation in the cohort study (n=544), as of January 1, 2018.

Demographic and clinical characteristics			n (%)
Male sex			297 (55)
Age at the time of the study (years)^a^			32.5 (25.4-38.5, 18.3-56.0)
**Age category at the time of the study (years)**			
	<20		30 (6)
	20-29		197 (36)
	30-39		207 (38)
	>39		110 (20)
Age at diagnosis (years)^a^			6.7 (3.1-12.5, 0.1-17.5)
**Age category at diagnosis (years)**			
	<5		207 (38)
	5-9		134 (25)
	10-14		149 (27)
	15-19		54 (10)
Time since diagnosis (years)^a^			25.0 (17.9-32.0, 6.2-42.0)
**Time since diagnosis (years)**			
	5-10		31 (6)
	11-20		130 (24)
	21-30		220 (40)
	31-40		147 (27)
	>40		16 (3)
**ICCC-3^b^ cancer diagnoses**			
	I Leukemia		218 (40)
	II Lymphoma		118 (22)
	III CNS^c^		35 (6)
	IV Neuroblastoma		17 (3)
	V Retinoblastoma		11 (2)
	VI Renal tumor		38 (7)
	VII Hepatic tumor		6 (1)
	VIII Bone tumor		40 (7)
	IX Soft tissue sarcoma		33 (6)
	X Germ cell tumor		8 (2)
	XI&XII Other rare tumors^d^		20 (4)
**Era of treatment**			
		1976-1985	136 (25)
		1986-1995	199 (36)
		1996-2005	156 (29)
		2006-2012	53 (10)
**Risk group**			
		High-risk^e^	300 (55)
		Standard-risk^f^	244 (45)
Any radiation therapy			213 (39)
Any surgery			329 (61)
Any chemotherapy			531 (98)
Hematopoietic stem cell transplantation			28 (5)

^a^median (IQR, range).

^b^ICCC-3, International Classification of Childhood Cancer third edition.

^c^CNS, central nervous system.

^d^including Langerhans cell histiocytosis, other malignant epithelial neoplasms, malignant melanomas, and other or unspecified malignant neoplasms.

^e^anthracyclines and/or cardiac radiation.

^f^any chemotherapy other than anthracyclines.

## Discussion

This retrospective, single-center cohort study is investigating the prevalence of cardiac dysfunction and its risk factors in adult CCS and comparing conventional and speckle tracking echocardiography.

Few studies are comparable to ours. A single-center study in The Netherlands included 525 adult CCS who had been treated during 1966-1997 with anthracyclines, high-dose cyclophosphamide, high-dose ifosfamide, and/or cardiac radiation [8]. Conventional echocardiography was performed during 1996-2004 to measure LV shortening fraction, and subclinical cardiac dysfunction was observed in 27% of survivors during a median follow-up time of 15 years. Another hospital-based, single-center study at St. Jude Children’s Research Hospital in the United States assessed 1820 adult CCS exposed to anthracyclines and/or cardiac radiation during a median follow-up time of 23 years using conventional and speckle tracking echocardiography [7]. One-third of survivors with normal LVEF had abnormal longitudinal strain seen on speckle tracking echocardiography. Risk factors for pathological findings in conventional and speckle tracking echocardiography were treatment with anthracyclines and cardiac radiation. The modifiable cardiovascular risk factors of hypertension, abdominal obesity, dyslipidemia, and high fasting glucose were associated with abnormal longitudinal strain but not with LVEF, suggesting that speckle tracking might be more sensitive for detecting cardiac dysfunction.

Conventional and speckle tracking echocardiography have some strengths and weaknesses that need to be addressed. Until now, conventional echocardiography has been the most commonly used noninvasive imaging modality to quantify cardiac function [22]; therefore, most studies of cancer patients and survivors are based on LVEF. Impaired LVEF is a late sign of cardiac damage, and the chance of recovery is already small. Speckle tracking echocardiography might overcome this limitation as it has been shown to be superior to LVEF in diagnosing cardiac dysfunction and predicting cardiac mortality in patients with underlying cardiac disease [34,35] and in adults undergoing cancer therapy [36]. Also, there are studies that have investigated the evidence of strain measurements for the surveillance of chemotherapy-related cardiac dysfunction in adult cancer patients (SUCCOUR trial) [37]. However, for CCS, we do not know the prognostic value of speckle tracking echocardiography yet. Another limitation is that strain analysis depends on image quality, and is age-, sex-, vendor-, and software-dependent [38]. We try to overcome this by strictly adhering to our standard operating procedures and using reference values stratified by age and sex using the same vendor (GE Vivid E9) and software (Tomtec Imaging Systems) [24]. By using only one type of vendor equipment and software, we also avoid intervendor variability.

First among this study's limitations is that it is currently confined to a single center. However, the 9 centers treating children and adolescents with cancer in Switzerland collaborate closely and use uniform treatment protocols; we therefore expect that results from the University Hospital Bern are representative of all 9 centers in the country. We are also concerned that our study includes a heterogeneous group of CCS with relatively small numbers of patients in each subgroup defined by treatment exposure or type of cancer. We plan to overcome this limitation by expanding this study to a nationwide study that includes all 9 Swiss Pediatric Oncology Group clinics. Also, our study might be affected by survival bias, as the most severely affected childhood cancer patients and survivors have already died. This could underestimate the cardiotoxic effect of anticancer management. We will collect the number of cardiac deaths from the Swiss Mortality Statistics, and this information will be considered in the analysis and interpretation of the results.

Among this study's several strengths is our attempt to include the complete cohort of survivors treated at the University Children’s Hospital Bern since 1976 based on the database of the Childhood Cancer Registry. We repeat our invitation to nonresponders several times and ask about reasons for not participating. This reduces the potential for selection bias. Also, we link the Childhood Cancer Registry with the Swiss Federal Statistical Office to collect cardiac causes of death in ≥5-year survivors. Second, we have access to all treatment exposures based on actual chemotherapy road maps and are able to look into dose-response relationships. Third, we will continuously include new 5-year survivors and therefore gain knowledge about the risk of cardiac dysfunction in younger patients treated more recently. Finally, our study has been set up within routine survivorship follow-up care using the experience of a multidisciplinary and interdisciplinary team with close collaboration between pediatric and adult cardiology, pediatric hematology and oncology, and clinical epidemiology.

The preliminary results from this retrospective, single-center study suggest that a standardized cardiac assessment that is part of routine follow-up care done in collaboration between pediatric and adult specialists is feasible in Switzerland and widely accepted by survivors and health care providers. In the next step, we will include more Swiss centers in the study to provide standardized clinical follow-up care longitudinally to all CCS on a nationwide scale.

## Appendix

Multimedia Appendix 1 Peer review reports, Swiss Cancer League.

## Abbreviations

2D: 2-dimensional
3D: 3-dimensional
A: late diastolic left ventricular filling velocity
CCS: childhood cancer survivors
E: early diastolic left ventricular filling velocity
E/A: early to late left ventricle filling velocity
E’: mitral annular early diastolic velocity
E/e’: peak mitral flow velocity
GCS: global circumferential strain
GLS: global longitudinal strain
GRS: global radial strain
ICCC3: International Classification of Childhood Cancer third edition
LA: left atrial
LV: left ventricular
LVEF: left ventricular ejection fraction
RA: right atrium
RV: right ventricle
STS: 1-minute sit-to-stand test
TR: tricuspid regurgitation
