# Supplementary material for: Diagnosing Preclinical Cardiac Dysfunction in Swiss Childhood Cancer Survivors: Protocol for a Single-Center Cohort Study
Source: JMIR Res Protoc. 2020 Jun 10;9(6):e17724. doi: 10.2196/17724 (PMC7315371; doi:10.2196/17724)
Supplement: Multimedia Appendix 1 [file resprot_v9i6e17724_app1.pdf]

Scientific Office
Swiss Cancer League / Swiss Cancer Research
Effingerstrasse 40
P.O. Box 8219
CH-3001 Bern
t +41 31 389 91 16
f +41 31 389 91 62
scientific-office@swisscancer.ch
www.swisscancer.ch/research

Peer Review

**Project-Number**	**KLS-3886-02-2016**
**Principal Investigator**	**Prof. Dr. med. Nicolas von der Weid**
**Requested Sum**	**CHF 366'999.00**
**Reviewer Number**	**3**

Detailed Critical Comments

Note: The detailed critical comments will be passed on to the project applicant without divulging the name of the reviewer

Please provide from here on your detailed critical or supportive comments on following aspects of the proposed project:

* originality and innovative aspects or: social-economic impact and timeliness (for epidemiological
and related clinical studies)
* adequacy of proposed research methods
* feasibility of the PI's group (chance of successful realisations)
* past accomplishments (of the PI, the leading group as well as of the other groups)

Early and late cardiovascular events related to the treatment of malignancy are of considerable importance for several reasons: first, and the bases for this undertaking is to establish an understanding the incidence, severity, and timing of cardiac events that follow treatment of pediatric malignancy. Such knowledge will help to mitigate these events in the future, a goal of increasing relevance in view of the longer survival time among this group of patients. Events that had previously been thought to be rare are coming to the attention of both pediatric and adult cardiologists who then are charged with the management of late cardiac presentations that may be serious or lethal. This research will accumulate knowledge as to the true incidence, seriousness, and natural history of such events so that pediatric oncologists may include such possible late events as part of the risk-benefit analysis as they choose the most appropriate treatments for their patients. As the applicants point out, the present state of knowledge regarding such events often comes from pooled or secondary data with a host of compounding factors. While the group of patients identified through this proposal will be small, it nevertheless should provide data that is especially useful and not otherwise available. Along with improved oncologic survival, future cohorts of patients will benefit from preventive strategies and the application of early intervention.

The second hugely important aspect of this research relates to the cardiac status of the individual patients under review. The cardiac events following treatment is often a late finding, a fact that is true for abnormalities of contractility, valvar disorders, or pericardial disease. Early and intermediate stages of cardiac impairment may be totally asymptomatic at the onset and comes to the attention of the patient or his/her physician only after much damage has compromised cardiac reserves. It is now clear that early intervention may delay the presentation of symptomatic cardiac late effects, thereby having a huge impact on the quality of life of patients previously treated. The one-on-one encounter proposed in this grant should help identify these patients earlier than would be otherwise possible.

I might suggest to these investigators that those patients who cannot be located, either because they are lost to follow-up or because they have not survived to partake in this research be included in the final analysis to whatever extent is possible; while most will not have experienced a cardiac death, identifying those that have might be an important element.

Scientific Office
Swiss Cancer League / Swiss Cancer Research
Effingerstrasse 40
P.O. Box 8219
CH-3001 Bern
t +41 31 389 91 16
f +41 31 389 91 62
scientific-office@swisscancer.ch
www.swisscancer.ch/research

Peer Review

**Project-Number**	**KLS-3886-02-2016**
**Principal Investigator**	**Prof. Dr. med. Nicolas von der Weid**
**Requested Sum**	**CHF 366'999.00**
**Reviewer Number**	**4**

Detailed Critical Comments

Note: The detailed critical comments will be passed on to the project applicant without divulging the name of the reviewer

Please provide from here on your detailed critical or supportive comments on following aspects of the proposed project:

* originality and innovative aspects or: social-economic impact and timeliness (for epidemiological
and related clinical studies)
* adequacy of proposed research methods
* feasibility of the PI's group (chance of successful realisations)
* past accomplishments (of the PI, the leading group as well as of the other groups)

The project is to use a registry of children cancer survivors in order to better characterize the prevalence of cardio-vascular disease using medical exam and to investigate diagnostic tools and risk factors.

The 2 most original aspects of this research are:

- to use comprehensive medical exam to detect asymptomatic cardio vascular disease in adolescent or young adults after cancer and then to provide better assessment of the prevalence of this complication of cancer treatment.

- to enrich an internet-based tool (passport of care) to provide cancer survivors a summary of their treatment and a comprehensive plan for follow-up. Such a passport would bring a direct benefit to the patients participating into this cohort. In addition, this would be a unique opportunity to demonstrate the utility of this kind of tools for the community medicine.

Even if the objectives will probably be enriched as the data are collected, the level of details of the data should provide an exceptional tool for this type of research. This reviewer would encourage the authors to also plan the collection and storage of blood sample, which my turn out to cast a different light on risk factors.

The research methods make use of several resources developed in Switzerland during the last decades. Furthermore, the population of children is probably interested in participating to this cohort due to the direct benefic it can expect. There is then a logic in the scientific process. Last, the PI has a strong network of complementary expertises that should boost the project.

Therefore, even if the project is ambitious (to carry out complete exam of >850 children), this reviewer thinks that the PI has the possibility to acheive the objective of this project based on previous accomplishement and on the fact that they are starting from scratch, with the help of his various collaborators.

The level of publication as first or last authors of the PI is moderate but the acheivement in terms of organisation of the follow-up of cance survivors and as pediatrician are major and prove the ability of the PI to fulfill the expectations.

Scientific Office
Swiss Cancer League / Swiss Cancer Research
Effingerstrasse 40
P.O. Box 8219
CH-3001 Bern
t +41 31 389 91 16
f +41 31 389 91 62
scientific-office@swisscancer.ch
www.swisscancer.ch/research

Peer Review

**Project-Number**	**KLS-3886-02-2016**
**Principal Investigator**	**Prof. Dr. med. Nicolas von der Weid**
**Requested Sum**	**CHF 366'999.00**
**Reviewer Number**	**5**

Detailed Critical Comments

Note: The detailed critical comments will be passed on to the project applicant without divulging the name of the reviewer

Please provide from here on your detailed critical or supportive comments on following aspects of the proposed project:

* originality and innovative aspects or: social-economic impact and timeliness (for epidemiological
and related clinical studies)
* adequacy of proposed research methods
* feasibility of the PI's group (chance of successful realisations)
* past accomplishments (of the PI, the leading group as well as of the other groups)

The proposal by von der Weid and colleagues is an important one because it seeks to describe and validate the true prevalence of cardiovascular disease (CVD) in long-term childhood cancer survivors. They have assembled an excellent team of investigators who have the expertise to conduct such epidemiologic studies. When completed, this study has the potential to provide important information regarding screening and prevention in high risk childhood cancer populations. That being said, there are a few concerns:

1) The investigators are quite optimistic in their assertion that they will be able to recruit and perform all cardiovascular assessments within the allotted time period. This is an ambitious plan, and there is no indication that the investigators have the track record to perform these types of in-person assessments in the time described. Typically, investigators reference previous efforts where in-person assessments have been performed – something along the lines of pilot feasibility studies. This would go a long way towards validating their proposed approach.

2) Moreover, a participation rate of 70% is based on the response rate from questionnaires. The overall participation rate is likely to be substantially less than the one for the SCCSS – especially if the investigators are asking individuals to come in to the centers for in-person assessments. Also, it is not clear if the 70% SCCSS participation rate was achieved within a short time period (as proposed in the current study) or over many years (as most questionnaire-based studies do). Therefore, the investigators cannot rely on having 600 CCS participating in the current study. In their power calculations, there needs to be some sensitivity analyses to demonstrate that they would have adequate power to complete their study aims, even if they had a lower than expected participation rate.

Scientific Office
Swiss Cancer League / Swiss Cancer Research
Effingerstrasse 40
P.O. Box 8219
CH-3001 Bern
t +41 31 389 91 16
f +41 31 389 91 62
scientific-office@swisscancer.ch
www.swisscancer.ch/research

Peer Review

**Project-Number**	**KLS-3886-02-2016**
**Principal Investigator**	**Prof. Dr. med. Nicolas von der Weid**
**Requested Sum**	**CHF 366'999.00**
**Reviewer Number**	**6**

Detailed Critical Comments

Note: The detailed critical comments will be passed on to the project applicant without divulging the name of the reviewer

Please provide from here on your detailed critical or supportive comments on following aspects of the proposed project:

* originality and innovative aspects or: social-economic impact and timeliness (for epidemiological and related clinical studies)
* adequacy of proposed research methods
* feasibility of the PI's group (chance of successful realisation)
* past accomplishments (of the PI, the leading group as well as of the other groups)

The proposed project focuses on early stage cardiovascular disease (CVD) after treatment for a cancer diagnosed during childhood. Most previous studies investigated manifest CVD as side effect of childhood cancer therapy but little is known about the relationship between cancer treatment and asymptomatic CVD. With earlier diagnosis, treatment can be initiated sooner and prognosis improves. The research question is therefore timely and very interesting.

The project will be performed among the 882 patients included in the Swiss Childhood Cancer Survivor Study and treated in Bern. Patients will be invited to a cardiac follow-up examination (physical examination, electrocardiogram and echocardiography). Both symptomatic and non-symptomatic CVD will be recorded. Data on patient, tumor and treatment characteristics are already available in the SCCSS database or will be extracted from medical records. This regional project can later be expanded at national level.

The proposal is very well written. The methodology is clearly described and adequate. Power calculations are provided.They are based on a 70% participation rate, which may be overestimated.

The research team has considerable expertise in epidemiology, pediatric oncology, and cardiology and is highly qualified to conduct the research with success.

The budget is mainly requested for salaries (1 post-doc 30%, 1 PhD and 1 technician 40%) for 3 years. It is well justified and adequate.

Overall this is a relevant project which will increase the knowledge of CVD burden as late side-effect of cancer treatment in the childhood cancer survivor population in Switzerland.

Recommendation: funding

Scientific Office
Swiss Cancer League / Swiss Cancer Research
Effingerstrasse 40
P.O. Box 8219
CH-3001 Bern
t +41 31 389 91 16
f +41 31 389 91 62
scientific-office@swisscancer.ch
www.swisscancer.ch/research

Peer Review

**Project-Number**	**KLS-3886-02-2016**
**Principal Investigator**	**Prof. Dr. med. Nicolas von der Weid**
**Requested Sum**	**CHF 366'999.00**
**Reviewer Number**	7

Detailed Critical Comments

Note: The detailed critical comments will be passed on to the project applicant without divulging the name of the reviewer

Please provide from here on your detailed critical or supportive comments on following aspects of the proposed project:

* originality and innovative aspects or: social-economic impact and timeliness (for epidemiological and related clinical studies)
* adequacy of proposed research methods
* feasibility of the PI's group (chance of successful realisation)
* past accomplishments (of the PI, the leading group as well as of the other groups)

Innovation: Late effects after cancer in childhood are an important research issue as the survival is increasing. Several years after the cancer diagnosis the risk of a secondary cancer or cardiovascular events is much higher for children with a malignancy than for other young adults. Therefore, the investigation of late effects is important. The study proposed by Prof. Nicolas von der Weid is therefore relevant and timely. A similar project is currently carried out in Mainz.

Methods:

The study design is a cross sectional investigation nested in a prospective cohort study, Swiss childhood cancer survivor study. Children who have been diagnosed in the hospitals of Bern at an age lower than 20 years between 1976 and 2012 will be invited to have cardiologic investigation. The number of children who will be invited is estimated to be around 800 (taking into account that about 1400 patients were treated, some 426 have died and some may have been lost to follow-up) and taking into account a participation rate of 55%.

The methods are well described (and straightforward), rather standard but correct. The investigators are quite optimistic about the response rates (55%). Additionally, the time line is very ambitious but with all the experience childhood cancer registries have, the applicants should be able to do it as proposed. If patients are still living in the area of Bern, the participation rate may be realistic.
